# Clinico Hemato Biochemical Findings, Clinical Management, and Production Performance of Bovines with Late Pregnancy Indigestion (Type IV Vagal Indigestion)

**DOI:** 10.1155/2014/525607

**Published:** 2014-04-07

**Authors:** Syed Ashaq Hussain, Sanjeev Kumar Uppal, Naresh Kumar Sood, Shashi Kant Mahajan

**Affiliations:** ^1^Department of Veterinary Medicine, Guru Angad Dev Veterinary and Animal Sciences University, Ludhiana, Punjab 141004, India; ^2^Department of Veterinary Pathology, Guru Angad Dev Veterinary and Animal Sciences University, Ludhiana, Punjab 141004, India; ^3^Department of Veterinary Surgery and Radiology, Guru Angad Dev Veterinary and Animal Sciences University, Ludhiana, Punjab 141004, India

## Abstract

This prospective study was conducted on 15 animals (eight buffaloes and seven cows), diagnosed with late pregnancy indigestion. Ten buffaloes and 10 cows served as the control group. The animals were in advanced pregnancy and had partial or complete anorexia, reduced water intake, loss of defecation or scanty faecal output, and mild to moderate dehydration. Heart and respiration rates were increased and rumen motility was reduced. Five animals had persistent tympany and moderate distension of left abdomen, and two animals each had bilateral abdominal distension and papple shaped abdomen. Neutrophil and lymphocyte counts were significantly higher and lower than the control values. Total bilirubin, AST, total protein, globulin, BUN, glucose, and lactate were significantly higher, and chloride and calcium were significantly lower than the control values. Levels of ALP, GGT, albumin, creatinine, cholesterol, triglyceride, fibrinogen, fibrinogen ratio, sodium, potassium, phosphorus, and magnesium did not differ significantly from the control values. Rumen chloride concentration was higher than the reference range. Majority of animals were managed symptomatically until parturition. There was no effect on fetal survival or milk yield in current and subsequent lactation. So, late pregnancy indigestion causes clinical and hemato biochemical alterations which require special consideration when treating diseased animals.

## 1. Introduction

Hoflund [1], on the basis of his experimental study, concluded that injury to the vagus nerve is the main etiological factor for the production of chronic indigestion and hence coined the term “vagal indigestion.” He classified disturbances of stomach into four types: (i) functional stenosis between the reticulum and omasum with atony of the rumen and reticulum, (ii) functional stenosis between the reticulum and omasum with normal or hyperactive ruminal and reticular activity, (iii) permanent functional stenosis of pylorus with atony or retained activity of the reticulum, and (iv) incomplete pyloric stenosis. Gastrointestinal dysfunction due to advanced pregnancy is considered to be type IV vagal indigestion [2] and is termed as late pregnancy indigestion (LPI). LPI is the reversible abomasal motility/outflow disorder that results from compression of the abomasum or proximal portions of the small intestine by the gravid uterus [3]. The diagnosis of LPI is usually by excluding the other causes of vagal indigestion and amelioration of clinical signs after parturition. The literature about hematological and biochemical alteration in bovine late pregnancy indigestion is scarce. So the prospective present study was undertaken to investigate several aspects of blood biochemical profile in bovines with LPI. Another objective of the study was to determine treatment outcome, fetal survival, milk production in the current and subsequent lactation, and recurrence of the disease.

## 2. Materials and Methods 

### 2.1. Selection of Animals and Criteria for Diagnosis of LPI

The study protocol was performed in compliance with the institutional guidelines. All owners gave their consent for cattle and buffaloes to be included in the study and undergo the testing procedures. The present prospective study was conducted on eight buffaloes and seven cows, out of 268 clinical cases of gastrointestinal disorders (173 buffaloes and 95 cattle), presented at Large Animal Clinics of Teaching Veterinary Hospital, Guru Angad Dev Veterinary and Animal Sciences University (GADVASU), Ludhiana, India, during the study period from May 2009 to April 2011. In 13 animals, the diagnosis of LPI was made on the basis of clinical examination, presence of abomasal reflux, and spontaneous recovery after induced or natural parturition. In two animals diagnosis was established after clinical examination and necropsy examination. Before predicting the diagnosis other causes of gastrointestinal dysfunction were ruled out by physical examination and other ancillary tests. None of these animals showed evidence of other diseases (even on necropsy of two animals).

### 2.2. Signalment and Anamnesis

Data related to species, age, parity, duration of illness, and pregnancy status were recorded in all the animals. A detailed history of feed intake, water intake, rumination status, defecation, type of tympany, and symptoms of regurgitation, fever, and pain were noted in every case. Ten cows and ten buffaloes from the university dairy farm served as the control group.

### 2.3. Physical Examination

Each animal was subjected to general physical examination and special examination of gastrointestinal system. Each animal was thoroughly evaluated for its general condition (alert or depressed), hydration status, and signs of pain. The physical parameters (rectal temperature, heart rate, respiration rate, colour of mucous membrane, and muzzle status) were recorded at the time of presentation in resting animal. Special examination of gastrointestinal system included observation of abdominal contour, rumen consistency, rumen motility, palpation of xiphoid region and wither pinch test for eliciting signs of pain, auscultation and percussion of abdomen for presence of fluid in intestines or abdominal cavity, and rectal examination. Radiography of reticular area was carried out to rule out foreign body syndrome.

### 2.4. Hematological and Biochemical Parameters

Blood samples (2 mL) were collected aseptically from jugular vein in EDTA coated vials (Accuvete-PLUS, Quantum Biologicals Pvt. Ltd.). The whole blood, immediately after collection, was used for determination of hematological parameters by standard methods [4].

For biochemical analysis, blood samples were collected in acid-free vials without any anticoagulant. Serum was separated and transferred to a dry clean vial for storage at −20°C till further evaluation. For glucose estimation blood was collected in vials containing sodium fluoride. Blood samples (2 mL) were collected in sodium citrate coated vials (Accuvete Disposables) for fibrinogen estimation. VITROS DT-II Chemistry system (Ortho-Clinical Diagnostics, Johnson and Johnson Company) was used for estimation of biochemical parameters. Fibrinogen was estimated by heat precipitation method using hand held refractometer [5]. Fibrinogen ratio was calculated by subtracting the fibrinogen concentration from the total protein concentration and then dividing that value by total protein concentration [5].

Rumen liquor samples were collected using 16-gauge, 4-inch long needle inserted perpendicularly into the left paralumbar fossa. Rumen liquor samples were evaluated for pH [6]. Rumen chloride was estimated after filtering the rumen liquor through a double layer muslin cloth and then centrifuging, using Bayer's diagnostic kits (colorimetric method) with the help of Microlab Autoanalyser (Merck).

### 2.5. Treatment and Followup

Twelve cases were managed medically till natural parturition or death. Treatment included intravenous administration of 10 liters of normal saline for 3–5 days, one-dose calcium therapy (450 mL of MIFEX, Novartis India Limited, India), 200 mL of liquid POTKLOR (containing 3 g of potassium chloride for 3 days), and liver tonic (8–10 mL of injection Livadex, Virbac Animal Health, India) and 100 g charcoal (as antibloat agent) on daily basis. Out of 12 animals, two animals did not show response to treatment and their abdominal distension increased leading to labored breathing. On the second and third days, respectively, these two animals became recumbent and eventually died. Necropsy examination of these two animals ruled out other causes of gastrointestinal dysfunction. In both cases, the rumen and reticulum were distended with frothy contents compressing the diaphragm, abomasum was compressed cranially, and intestines were collapsed. The death was ascribed to respiratory failure due to compression of diaphragm caused by abdominal distension. Rumenotomy was done in one animal, because the animal showed regurgitation at the time of presentation. Removal of ingesta from the rumen allowed some remission of symptoms, but 12 hours later, the animals again showed signs of abdominal distress and hence parturition was induced. Parturition was induced in another two animals (had severe abdominal distension), by intramuscular injection of dexamethasone (20 mg). To relieve the abdominal distension, rumen trocarization was done in these two animals.

Ampicillin (22 mg/Kg body weight) and enrofloxacin (7.5 mg/Kg BW) twice daily were administered to the animals with inflammatory leukogram, for 5 days. All the owners were telephonically contacted at regular intervals (every 15 days till 2 months, then at regular interval of three months) for a period of two years (i.e., up to 2012). The owners were questioned about the general health status of the animals and the born calves, any effect of milk yield in current and subsequent lactation, and recurrence of disease in the next pregnancy.

### 2.6. Statistical Analysis

All quantitative data were described as mean ± standard error. The comparison between control and diseased group was done by using Student's *t*-test. Significance was set at *P* ≤ 0.05 and *P* ≤ 0.01.

## 3. Results 

All the animals diagnosed for LPI were in advanced pregnancy (cows >8.5 months and buffaloes >9 months). The animals were 3–9-year-old (mean = 6.87 ± 0.61 years, median = 8 years) females and duration of illness was 2–18 days (mean = 7.4 ± 4.52 days, median = 5 days). The animals were expected to parturate in 3–22 days. The historical findings and physical examination and rectal examination parameters are presented in Tables 1 and 2. These animals had partial or complete anorexia and reduced water intake. Complete loss of defecation was seen in 6 cases and the rest of the animals had scanty faecal output. Seven and three animals had history of fever and abdominal pain, respectively. Five cases had persistent tympany while two animals had single episode of tympany. Eight animals were dull and depressed at the time of presentation while seven animals appeared alert. Conjunctival mucous membrane was congested in seven and pale in two animals. Mean heart rate (84.13 ± 4.5/minute) and respiration rate (37.33 ± 2.9/minute) were increased and all animals were mildly to moderately dehydrated. Rumen was atonic in three, hypomotile in eight, and hypermotile in two cases, and rumen consistency was mushy or doughy. Five animals had moderate distension of left paralumbar fossa, two animals had papple shaped abdomen, and two animals had bilateral abdominal distension. Perrectal examination revealed viable fetuses in all the cases. Radiography of reticular area revealed no abnormality.

The significant variations of hematological and biochemical parameters from respective control values are presented in Table 3. There were no significant differences from control values with respect to hemoglobin, packed cell volume (PCV), and total white blood cell (WBC) count. Neutrophil (*P* ≤ 0.01) and lymphocyte (*P* ≤ 0.05) counts were significantly higher and lower than the control value, respectively. Hematological analysis revealed neutrophilia in 11 and neutrophilic leukocytosis in three animals. The toxic changes in neutrophils were mild to moderate in 6 cases and severe in one case. Left shift was mild to moderate in five cases and marked in two cases. Total bilirubin (*P* ≤ 0.01), aspartate aminotransferase (AST) (*P* ≤ 0.01), total protein (*P* ≤ 0.05), globulin (*P* ≤ 0.01), blood urea nitrogen (BUN) (*P* ≤ 0.05), glucose (*P* ≤ 0.01), and lactate (*P* ≤ 0.01) were significantly higher than control values while chloride (*P* ≤ 0.01) and calcium (*P* ≤ 0.01) were significantly lower than the control values. Although alkaline phosphatase (ALP) and gamma glutamyltransferase (GGT) were higher than the reference values, they did not differ significantly from control value. Potassium and phosphorus were lower than reference values but did not differ significantly from control values. In all the animals, rumen pH was within normal reference range of 6.2–7.2, while rumen chloride concentration was higher than the standard reference range (<30 mEq/L).

Out of 12 animals, managed medically, two died within 3 days. Out of these 12, the 10 survived animals gave birth to the healthy calves without any calf mortality. In majority of the animals, there was no effect on milk production in the current and subsequent lactation. Recurrence of the disease in subsequent pregnancy was recorded in one animal only. As per the owner of this animal, the milk yield in the subsequent lactation was less than the expected. Three animals (in which parturition was induced, induced after rumenotomy in one animal) conceived and parturated without any complication. So, in total, thirteen animals made an uneventful recovery after a period of 5–27 days.

## 4. Discussion 

To the authors best knowledge this is the first study to describe detailed hematobiochemical findings in late pregnancy indigestion of cattle and buffaloes. The indigestion in advanced pregnancy may be attributed to compression of intestines by the gravid uterus resulting into functional pyloric stenosis [7]. Motility of gastrointestinal tract in laboratory animals is said to be affected by pregnancy and is believed to be hormonally mediated [8]. So another possible factor in the pathogenesis of LPI could be the hormonal changes of pregnancy. The consistent owner complaints were scanty or complete loss of defecation and anorexia or inappetance in advanced pregnancy. The common clinical signs were similar to those reported for pyloric stenosis [3, 7, 9] and were consistent with gastrointestinal dysfunction. Although papple shaped abdomen is thought to be a characteristic sign of vagal indigestion [2], it was observed in only two cases. Similar to present findings, Behl et al. [9] also observed that papple shaped abdomen was not a consistent finding in vagal indigestion of buffaloes. Although the animals showed response to treatment in terms of feed intake and faecal output, complete clinical recovery was observed only after the natural or induced parturition, which confirmed the diagnosis of LPI. Further the diagnosis was supported by an increased chloride concentration in rumen fluid and reduced chloride and potassium concentrations in blood, indicating abomasal reflux.

Hematological results were compatible with an inflammatory process that was attributable to inflammatory response to gastrointestinal stasis leading to absorption of toxins into circulation [6]. Decreased lymphocyte count could be due to release of corticosteroids as a result of stress [4]. In functional pyloric stenosis, increase in the total white blood cell count with an increased percentage of either band cells or polymorphonuclear cells or both has been reported earlier [10, 11]. Clinically the signs of dehydration were obvious but PCV value was within normal reference range. It was in accordance with the previous finding [9] that, in vagal indigestion, PCV may remain within the normal range even though clinical signs of dehydration are present.

Hepatic damage due to absorption of toxins from the putrified rumen ingesta, due to gastrointestinal stasis, may be the cause for higher liver enzyme activities [6]. Absorption of toxic products from rumen or alimentary tracts, starvation, and constipation leading to cellular disturbances of liver parenchyma have been reported to cause increased levels of plasma AST and total bilirubin [12]. The increase in total protein may be due to release of some acute phase proteins and increased globulin concentration in response to inflammation, stress, or dehydration [12]. The concentration of plasma proteins in vagal indigestion has been reported to be either normal [9, 10] or increased [7, 11]. The normal value of fibrinogen ratio indicated that there was no realistic increase in fibrinogen. Dehydration along with decreased lactate uptake due to hepatic malfunction may be the causes for hyperlactatemia [13]. Hyperglycemia may be due to stress of gastrointestinal dysfunction and advanced pregnancy leading to adrenocorticosteroid release, which has glycogenolytic effect. Abomasal reflux (indicated by increased rumen chloride concentration) could be the cause for dehydration, hypochloremia, hypokalemia, and azotemia [9, 10]. Another cause of dehydration and electrolyte disturbance may be the loss of water and electrolytes due to salivary secretions. Hypocalcaemia and hypophosphatemia may be due to less assimilation of feed materials [14]. Daniel [15] reported both reduced rumen and abomasal motilities were similarly reduced in hypocalcaemia due to general effect of depression of levels of ionized calcium on smooth muscle contractibility. Similarly, reduced calcium level in the present study may have contributed to decreased ruminal motility and hence loss of defecation or scanty faeces. Similar to present findings, van Metre et al. [3] have also observed hypocalcaemia, hypochloraemia, and hypokalemia in indigestion of late pregnancy.

The literature regarding treatment of LPI is scarce. So in the present study animals were managed by symptomatic treatment, mainly to reduce inflammatory response and abdominal distension and to correct the electrolyte deficit. The electrolyte imbalances were assessed to avoid complications associated with hypokalemia, hypochloremia, and hypocalcaemia. Results of this study suggest that prognosis for LPI is good. So, in order to reduce economic losses due to calf mortality, it is recommended that animals with LPI could be managed medically till parturition.

## 5. Conclusions

Amelioration of clinical signs after parturition (natural or induced) was considered as a definitive diagnostic for LPI. LPI caused significant hematobiochemical alterations which require greater attention in treatment of affected animals. So these hematobiochemical changes should be taken into consideration while treating LPI in bovines. There was no effect on fetal survival in medically managed cases. So such cases could be managed medically, till parturition, in order to reduce the losses due to calf mortality, which may occur by induction of parturition. The prognosis of the disease was good and recurrence rate was very low.

## Figures and Tables

**Table 1:** History and clinical observations of 15 animals with late pregnancy indigestion.

Characteristic	Finding	Number of animals
Feed intake	Reduced	3
	Absent	12

Water intake	Normal	6
	Reduced	9

History of abdominal pain	Present	3
	Absent	12

History of fever	Present	7
	Absent	8

History of tympany	Persistent	5
	Once	2
	Absent	8

Defecation	Scanty	9
	Absent	6

General expression	Alert	8
	Depressed	7

Visual findings	Mild to moderate left side distension	5
	Severe left side distension	6
	Bilateral distension	2
	Papple shaped abdomen	2

Mucous membrane	Normal	6
	Congested	7
	Anemic	2

Dehydration	Mild	10
	Moderate	5

Temperature	Normal (99–102°F)	11
	Increased (>102°F)	4

Heart rate/minute	Normal (60–80)	8
	Slightly to moderately increased (81–100)	4
	Greatly increased (>100)	3

Respiration rate/minute	Normal (15–25)	3
	Increased (>26)	12

Rumen motility/2 minutes	Normal (3/2)	2
	Reduced (<3/2)	8
	Absent	3
	Increased (>3/2)	2

Treatment	Medicinal till natural parturition or death	12*
	Rumenotomy followed by induced parturition	1
	Induction of parturition	2

*Two animals died within three days.

**Table 2:** Rectal exploration findings in 15 animals with late pregnancy indigestion.

Characteristic	Finding	Number of animals
Faecal quantity in rectum	Reduced	3
	Scanty	10
	Absent	2

Faecal consistency	Normal	6
	Faeces absent	2
	Pelleted	1
	Hard	2
	Loose	3
	Pasty	1

Presence of mucous	Absent	14
	Mucous with faeces	1

Hindered hand movements	No	2
	Yes	13

Rectal mucosa	Normal	12
	Sticky	3

Rumen size	Normal	7
	Mild to moderate distension	6
	Severe distension	2

Rumen consistency	Mushy	7
	Doughy	7
	Hard	1

Intestines	Normal	14
	Mild to moderate distension	1

**Table 3:** Hematology and biochemical parameters of 15 animals with late pregnancy indigestion and control group animals (mean ± S.E.M).

Measurement	Late pregnancy indigestion	Control group
Hb (g/dL)	11.09 ± 0.53	10.65 ± 0.46
PCV (%)	34.38 ± 1.94	32.72 ± 1.69
WBC (/*μ*L)	11450 ± 278.07**	9733 ± 1106.8
Neutrophils (/*μ*L)	6983 ± 1147.56*	3636 ± 993.8
Lymphocytes (/*μ*L)	4430 ± 434.6	5860 ± 376.38
Monocytes (/*μ*L)	67.17 ± 32.2	61.0 ± 27.97
Eosinophils (/*μ*L)	20.27 ± 51.72	1.60 ± 0.34
Total bilirubin (mg/dL)	1.23 ± 0.48**	0.09 ± 0.42
AST (U/L)	237.67 ± 74.61**	101.05 ± 64.80
ALKP (U/L)	113.60 ± 29.96	89.55 ± 25.99
GGT (U/L)	67.73 ± 29.70	39.15 ± 25.72
Total protein (g/dL)	7.95 ± 0.28**	7.47 ± 0.24
Albumin (g/dL)	3.43 ± 0.15	3.41 ± 0.13
Globulin (g/dL)	4.52 ± 0.23**	4.07 ± 0.20
BUN (mg/dL)	30.20 ± 6.19*	17.20 ± 5.36
Creatinine (mg/dL)	2.34 ± 0.68	1.19 ± 0.59
Glucose (mg/dL)	103.69 ± 13.68**	60.10 ± 11.84
Lactate (mmol/L)	6.48 ± 1.25**	1.51 ± 1.08
Cholesterol (mg/dL)	71.49 ± 15.69	84.40 ± 13.59
Triglyceride (mg/dL)	35.50 ± 5.23	27.75 ± 4.53
Fibrinogen (g/dL)	0.39 ± 0.11	0.33 ± 0.96
Fibrinogen ratio	25.35 ± 2.57	32.30 ± 2.97
Sodium (mmol/L)	134.47 ± 2.87	139.00 ± 2.49
Potassium (mmol/L)	3.61 ± 0.3	4.15 ± 0.25
Chloride (mmol/L)	89.27 ± 3.39**	101.70 ± 2.94
Calcium (mg/dL)	7.21 ± 0.40**	9.30 ± 0.35
Phosphorus (mg/dL)	5.51 ± 0.48	5.88 ± 0.42
Magnesium (mg/dL)	2.98 ± 0.31	2.36 ± 0.27
Rumen chloride (mEq/L)	43.82 ± 3.61	—

^∗,∗∗^ Indicate significant difference from control group at *P* ≤ 0.05 and *P* ≤ 0.01, respectively.
